# Chemical Profiling, In Silico and In Vitro Studies to Identify Potential CDK2 and mTOR Inhibitor From *Selaginella inaequalifolia* (Hook. & Grev.) Spring Ethanolic Extracts

**DOI:** 10.1002/cbdv.202502119

**Published:** 2025-11-29

**Authors:** Johnson Marimuthu Alias Antonysamy, Sivaraman Arumugam, Robin Charles Antony Arulraj, Anne Wincy Jacob Thomas, Henrique D. M. Coutinho

**Affiliations:** ^1^ Centre for Plant Biotechnology, Department of Botany St. Xavier's College (Autonomous) Palayamkottai India; ^2^ Maria Research Foundation Alagar Nagar Tirunelveli India; ^3^ Department of Computer Science Sarah Tucker College (Autonomous), Perumalpuram Tirunelveli India; ^4^ Department of Biological Chemistry Regional University of Cariri Crato Brazil

**Keywords:** fern allies, in silico, lycophytes, phytoprofile, GC–MS

## Abstract

The current study is aimed to reveal the phytoprofile of *Selaginella inaequalifolia* (Hook. & Grev.) Spring using GC–MS and predict the drug properties, toxicity, biological properties of *S. inaequalifolia* ethanolic extracts (*Si*EE) using in silico methods and in vitro toxicity assays, namely, MTT and BSLB assay. A total of 27 compounds are identified from *Si*EE with varied physicochemical properties. Various biological properties of the identified compound are predicted based on online PASS prediction. The ADME and toxicity profile analysis identified pentadecanoic acid, 13‐methyl‐, methyl ester as a strong CDK2 and mTOR inhibitor, suggesting anticancer potential. A dose‐dependent toxicity and cytotoxicity are observed. The brine shrimp lethality assay (LC_50_: 274.26 mg/mL) indicated low cytotoxicity, while MCF‐7 breast cancer cell line studies (IC_50_: 42.49 µg/mL) showed promising anticancer activity. These findings support *S. inaequalifolia* as a potential source of therapeutic agents, warranting further molecular and clinical investigations.

## Graphical abstract

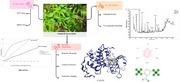

## 1 Introduction

In Asia and Africa, 80% of peoples are depending on the traditional herbals for their medicinal requirements [1]. In the modern world, plant extract is eco‐friendly in nature, and are used as the source of insecticides, pesticides, larvicidal, wound healing, antibacterial, antioxidant, anti‐inflammatory, antidiabetic, ant‐cancer, and so forth [2, 3, 4, 5, 6, 7, 8]. Pteridophytes, including ferns and horsetails, have gained attention for their cytotoxic properties, particularly in cancer research, which have been shown to inhibit cell growth and induce apoptosis in cancer cells [9, 10]. Species like *Cyathea* have demonstrated significant cytotoxicity against MCF‐7 cancer cell lines, suggesting their potential for anticancer therapy [11]. In vitro assays, including brine shrimp lethality and MTT assays are commonly used to evaluate the cytotoxic effects of pteridophytes genus like *Phlebodium*, *Cyathea*, *Selaginella*, and *Pteris* extracts [12, 13]. These studies highlight the promising role of pteridophytes as natural sources of cytotoxic agents with selective activity against cancer cells.

The genus *Selaginella* belongs to Selaginellaceae family with 700–750 species. Several species of *Selaginella* are used as medicines and food from pre‐historic period [14]. *Selaginella* is used as the traditional medication for wounds, childbirth, problems with menstruation, skin diseases, headaches, fevers, urinary tract infections, liver disease, tumors, osteoarthritis, and bone fractures etc. [15]. Various metabolites like lignans [16], terpenoids [17], triterpenoids [23], alkaloids [18], flavonoids [63] are reported from several species of *Selaginella*, which have shown several kinds of biological measures such as antioxidant [3, 19, 20], immunomodulatory [19], antimicrobial [21, 22, 23], antibacterial [24], antifungal [25], anticancer [26], antidiabetic [27], cytotoxicity [12]. Nine derivatives of special chemicals known as selaginellins are present in species of *Selaginella* contains alkynyl phenol and *p*‐quinone methide functional groups and isolated some compounds like selaginisoquinoline and 3 ethoxy selaginellins [28], 16 compounds are isolated including two new secondary metabolites from *Selaginella doederleinii* [29]. New two flavone glucosides namely, 7‐*O*‐(β‐glucopyranosyl (1→2)‐[β‐glucopyranosyl(1→6)]‐β‐glucopyranosyl)flavone‐3′,4′,5,7‐tetraol and 7‐*O*‐(β‐glucopyranosyl(1→2)‐[β‐glucopyranosyl(1→6)]‐β‐glucopyranosyl)flavone‐4′,5,7‐triol are isolated, two new biflavonoids 2,3‐dihydroflavone‐5,7,4′‐triol‐(3′→8″)‐flavone‐5″,6″,7″,4‴‐tetraol and 6‐methylflavone‐5,7,4′‐triol‐(3′→O→4‴)‐6″‐methylflavone‐5″,7″‐diol, two new lignans (7′E)‐3,5,3′,5′‐tetramethoxy‐8:4′‐oxyneolign‐7′‐ene‐4,9,9′‐triol and 3,3′‐dimethoxylign‐8′‐ene‐4,4′,9‐triol together with two known monolignans, four known lignans, and four known bioflavonoids are isolated from *Selaginella moellendorfii* (Wu et al. 2011) [30]. Based on the pharmacological qualities, molecular systematic and micro morphological characteristics, *Selaginella* is an interesting and little‐studied genus [31].

Target proteins are primarily selected based on their established role in disease pathogenesis and their regulations. For example, CDKs are critical regulators of cell cycle progression, DNA replication, and transcription, and their dysregulation is strongly implicated in cancer development [32]. Proteins that act as key nodes in disease‐related signaling pathways or metabolic networks are prioritized because inhibiting their activity can effectively disrupt pathological processes [33]. In the present study, cyclin‐dependent kinase 2 (CDK2) (PDB ID: 4GCJ) and serine/threonine‐protein kinase mTOR (PDB ID: 4JSX) are selected.

Several reports have demonstrated strong binding affinities of *Selaginella*‐derived compounds through molecular docking for instance, *S. doederleinii* extract having good binding affinity in diosgenin with GLUT1 and LDHA (−11.8 and −9.6 kcal/mol, respectively) [34], and robustaflavone with α‐glucosidase (−11.33 kcal/mol) (Gao et al. 2024) [35]. *Selaginella bryopteris* having a stability of amentoflavone in complex with MAPK1 and MAPK14 proteins are confirmed through molecular dynamics simulations and MM‐PBSA analysis [60]. These computational efforts exhibit notable limitations. Most existing studies focus on individual compounds rather than complete extract profiling, thereby overlooking possible synergistic effects among multiple bioactive constituents, which may be insufficient to capture long‐term stability and conformational dynamics of protein–ligand complexes. Furthermore, current research tends to emphasize isolated therapeutic targets without developing a comprehensive structure–activity relationship across different *Selaginella* species and their diverse phytochemical compositions. Another major shortcoming is no report on the chemical constituents and in silico studies on *Selaginella inaequalifolia* (Hook. & Grev.) Spring ethanolic extracts. To fill the gap, the current study is aimed to reveal the phytoprofile of *S. inaequalifolia* using GC–MS and predict the drug properties, toxicity, biological properties of *S. inaequalifolia* ethanolic extracts (*Si*EE) using in silico methods and in vitro toxicity assays, namely, MTT and BSLB assay.

## 2 Materials and Methods

### 2.1 Collection of Plants

Healthy, disease‐free *S. inaequalifolia* (Hook. & Grev.) Spring collected from Kakachi, Kothayar, Tirunelveli Hills, Western Ghats, South India on January 26, 2021. The plants are identified by Dr. M. Johnson Curator, Centre for Plant Biotechnology Herbarium, St. Xavier's College (Autonomous) based on the “Pteridophyte Flora of the Western Ghats, South India” by Manickam and Irudayaraj [36]. Herbarium specimen is prepared at the collection site itself and the voucher specimen (CPBH 1303) is deposited in the Centre for Plant Biotechnology Herbarium (CPBH), Palayamkottai, Tamil Nadu, India.

### 2.2 Preparation of Extracts

The collected whole plants of *S. inaequalifolia* are thoroughly washed with tap water followed by distilled water. The washed whole plants of *S. inaequalifolia* are blotted on the blotting paper and spread out at room temperature in shade to remove the excess water contents. The shade dried whole plants of *S. inaequalifolia* are ground to fine powder using mechanical grinder. The powdered samples are stored in refrigerator for further use. Thirty grams of powdered materials of *S. inaequalifolia* are extracted with 180 mL of ethanol using Soxhlet extractor for 8 h at a temperature not exceeding the boiling point of the solvent. The *Si*EE are filtered using Whatman filter paper (No. 1) and then concentrated in vacuum at 40°C using rotary evaporator. The obtained residues of *Si*EE are stored in a freezer until further tests.

### 2.3 GC–MS Analysis

To reveal the chemical constituents, present in the *Si*EE, gas chromatography–mass spectrometry (GC–MS) analysis is performed using the Clarus 500 GC–MS (PerkinElmer). Two microliters of *Si*EE is injected for GC–MS analysis [37, 58]. The Clarus 500 GC used in the analysis employed a fused silica column packed with Elite‐1 (100% dimethyl poly siloxane, 30 nm × 0.25 nm ID × 1 µm df) and the compound constituents are separated using helium as carrier gas at a constant flow of 1 mL/min. Two microliters *Si*EE injected into the instrument is detected by the Turbo gold mass detector (PerkinElmer) with the aid of the Turbo mass 5.1 software. During the 36th minute of GC extraction process, the oven is maintained at a temperature of 110°C with 2 min holding. The injector temperature is set at 250°C (mass analyzer). The different parameters involved in the operation of Clarus 500 MS, are also standardized (inlet line temperature: 2000°C; source temperature: 2000°C). Mass spectra are taken at 70 eV; a scan interval of 0.5 s and fragments from 45 to 450 Da. The MS detection is completed in 38 min. The identified compounds biological activities are predicted using PASS.

### 2.4 ADME and Toxicity Prediction

ADMET is employed to study the in silico ADMET and the toxicity properties of the identified chemical compounds of *Si*EE. To reveal the in silico ADME and toxicity of chemical constituents identified from *Si*EE, namely, 2,5‐dihydroxyacetophenone, bis(trimethylsilyl) ether, cyclopentasiloxane, decamethylcyclopentasiloxane, oxazepam DITMS, hydroquinone 1,4‐benzenediol, 1‐hexadecene, methyl 3‐hydroxyl tetradecanoate, tetradecanoic acid, 3‐hydroxy‐, methyl ester, salicylaldehyde hydrazone benzaldehyde, 2‐hydroxy‐, hydrazine, α‐d‐glucopyranose, 4‐*O*‐α‐d‐galactopyranosyl lactose, α, 2‐propenoic acid, 3‐(4‐fluorophenyl)‐, ethyl ester, î‐*N*‐formyl‐l‐lysine, pentadecanoic acid, 13‐methyl‐, methyl ester, 4‐cyclohepta‐2,4,6‐trienyl‐benzoic acid, 5,6‐dimethoxy phthalaldehydic acid, the smile format are retrieved from NCBI PubChem and submitted to the Swiss ADME (http://www.swissadme.ch/). Swiss ADME online server analyzes various parameters including physicochemical properties, medicinal chemistry, pharmacokinetics, water solubility of the identified compounds of *Si*EE. Similarly, toxicity factors such as acute inhalation, acute oral, acute dermal toxicity, eye irritation and corrosion, skin sensitization, skin irritation and corrosion of the identified compounds of *Si*EE are also predicted using online tool STOP TOX (https://stoptox.mml.unc.edu/) [38, 39].

### 2.5 Preparation of Ligands

The 3D SDF file format of the identified ligands of *Si*EE, namely, 2,5‐dihydroxyacetophenone, bis(trimethylsilyl) ether, cyclopentasiloxane, decamethyl, oxazepam ditms, hydroquinone 1,4‐benzenediol, cyclodecasiloxane, 1‐hexadecene, methyl 3‐hydroxyl tetradecanoate tetradecanoic acid, 3‐hydroxy‐, methyl ester, salicyl aldehyde hydrazone benzaldehyde, 2‐hydroxy‐, hydrazine, α‐d‐glucopyranose, 4‐*O*‐α‐d‐galactopyranosyl lactose, α, 2‐propenoic acid, 3‐(4‐fluorophenyl)‐, ethyl ester, î‐*N*‐formyl‐l‐lysine, pentadecanoic acid, 13‐methyl‐, methyl ester, 4‐cyclohepta‐2,4,6‐trienyl‐benzoic acid, and 5,6‐dimethoxyphthalaldehydic acid are downloaded from PubChem (https://pubchem.ncbi.nlm.nih.gov/) database for in silico docking analysis. The in silico docking analysis is performed between the identified compounds of *Si*EE and selected proteins, namely, CDK2 (PDB ID: 4GCJ), serine/threonine‐protein kinase mTOR (PDB ID: 4JSX).

### 2.6 Preparation of Proteins

The targeted proteins, namely, CDK2 (PDB ID: 4GCJ) and serine/threonine‐protein kinase mTOR (PDB ID: 4JSX) are downloaded from RCSB (Research Collaboratory for Structural Bioinformatics) Protein Data Bank (PDB) (https://www.rcsb.org/) in PDB format.

### 2.7 Molecular Docking Analysis

The identified compounds/ligands of *Si*EE are docked against the protein CDK2, serine/threonine‐protein kinase mTOR using CB‐DOCK 2 (http://183.56.231.194:8001/cb‐dock2/php/blinddock.php#job_list_load) [40, 62].

### 2.8 Cytotoxic Activity—MTT Cell Proliferation Assay Cell Line and Culture

To validate the in silico observation, the toxicity and cytotoxicity studies are performed for *Si*EE against brine shrimp and MCF cell lines. The cell line of MCF‐7 (human breast carcinoma) is obtained from National Centre for Cell Science, Pune, India and the experiments are performed in Amala Cancer Research Centre, Thrissur, Kerala, India. The cells are cultured in a growth medium (DMEM, PH 7.4), supplemented with 10% FBS and antibiotics, penicillin (100 units/mL) and streptomycin sulfate (100 µg/mL).

### 2.9 MTT Assay

The cytotoxicity of *Si*EE against human breast carcinoma (MCF‐7) is determined by the MTT (3‐[4,5‐methylthiazol‐2‐yl]‐2,5‐diphenyl‐tetrazolium bromide) assay [41]. The cells are seeded into wells of microtiter plate (96 well) at 3 × 10^3^ cells per well with 100 µL of DMEM growth medium. It is then incubated for 24 h at 37°C under 5% CO_2_ in a humidified atmosphere. Later, the medium is removed and fresh growth medium containing different test doses of *Si*EE (12.5, 25, 50, 100, and 200 µg/mL) are added. Five wells are included in each concentration. After 3 days of incubation at 37°C under 5% CO_2_, the medium is removed. Twenty microliters of 5 mg/mL MTT (pH 4.7) is added per well and cultivated for another 4 h, the supernatant fluid is removed. One hundred microliters of DMSO is added per well and shaken for 15 min. The absorbance at 570 nm is measured with a UV‐spectrophotometer, using wells without cells as blanks. All the experiments are performed in triplicates. The absorbance of untreated cells is considered 100%. The IC_50_ value is determined graphically (Scatter Regression) by using MS EXCEL. The conventional anticancer drug, adriamycin is used as a positive control. The inhibition of cell growth is calculated as percent anticancer activity using the following formula:

$$\begin{array}{ccc} & & \%\;\mathrm{of}\;\mathrm{Cell}\;\mathrm{inhibition}\\ & & \;=100-\left(\mathrm{sample}\;\mathrm{absorbance}/\mathrm{control}\;\mathrm{absorbance}\right)\\ & & \;\times \;100 \end{array}$$

### 2.10 Brine Shrimp Lethality Bioassay

Cytotoxic activity of *Si*EE is evaluated using brine shrimp lethality bioassay method [42]. About 1 g of *Artemia salina* cysts is aerated in 1 L capacity glass jar containing filtered seawater. The air stone is placed in the bottom of the jar to ensure complete hydration of the cysts. After 24 h incubation at room temperature (25°C–29°C), newly hatched free‐swimming nauplii are harvested from the bottom outlet. As the cysts capsules are floated on the surface, this collection method ensured pure harvest of nauplii. The freshly hatched free‐swimming nauplii are used for the bioassay. Thirty clean test tubes are taken, of which 25 tubes are used for the samples with five different concentrations, namely, 100, 200, 300, 400, and 500 mg/mL and five tubes for control. With the help of a Pasteur pipette, 20 nauplii are transferred to each tube containing various concentrations of *Si*EE. Five replicates are made for each concentration and a control DMSO is also maintained. The standard plumbagin is used as positive control. The setup is allowed to remain for 24 h under constant illumination. After 24 h, the dead nauplii are counted with a hand lens. Using the recorded observations, LC_50_, LC_90_, and chi square values are also calculated.

### 2.11 Statistical Analysis

For the cytotoxicity analysis using MCF cell lines, the IC_50_ value is determined graphically (scatter diagram) by using MS EXCEL, the concentration of *Si*EE and adriamycin is taken in the *X*‐axis and the percentage of growth inhibition is taken in the *Y*‐axis. For the toxicity analysis using BSLB, LC_50_, LC_90_, and chi square values are calculated using SPSS.

## 3 Results

**FIGURE 1:**
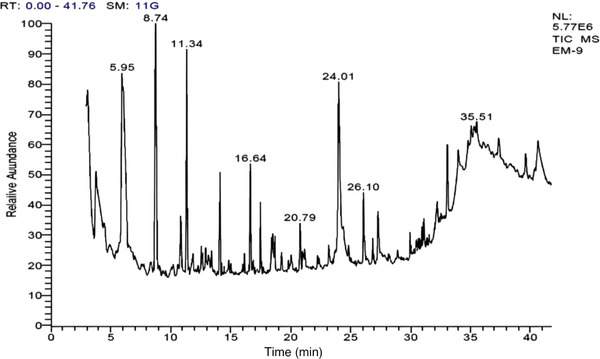
GC chromatogram of *Selaginella inaequalifolia* ethanolic extracts (*Si*EE).

A total of 27 compounds are identified from *SiEE* with varied retention time (RT) and their structures are identified based on mass spectrometry (Figure 1). The identified compound bioactivities are predicted based on online PASS prediction (Table 1).

**TABLE 1:** Chemical constituents of *Selaginella inaequalifolia* ethanolic extract and their biological activities.

Name of the identified compound	RT	Peak area%	Mol. formula	Mol.wt.	PASS predicted biological activities
Octaethyleneglycolmonododecylether	3.10	2.64	C_28_H_58_O_9_	538.8	Limulus clotting factor B inhibitor, styrene‐oxide isomerase inhibitor, muscle relaxant, acyl‐CoA hydrolase inhibitor, Omptin inhibitor
2,5‐Dihydroxyacetophenone, bis(trimethylsilyl)ether	3.77	2.61	C_14_H_24_O_3_Si_2_	296.51	Antiparasitic, benzoate‐CoA ligase inhibitor, acetylcholine neuromuscular blocking agent, Ornithine cyclodeaminase inhibitor
Cyclopentasiloxane, decamethylcyclopentasi Loxane	5.93	8.39	C_10_H_30_O_5_Si_5_	370.77	Phobic disorders treatment, chloride peroxidase inhibitor, phospholipid‐translocating ATPase inhibitor, Pterindeaminase inhibitor, chymosin inhibitor
Docosanoicacid,1,2,3‐propanetriylester docosanoin, tri‐	7.75	1.17	C_69_H_134_O_6_	1058.8	Thromboxane synthase stimulant, macrophage colony‐stimulating factor agonist, leukopoiesis stimulant, platelet aggregation stimulant, antisecretoric
Oxazepamditms	8.74	15.84	C_21_H_27_ClN_2_O_2_Si_2_	431.1	Anti‐inflammatory, interleukin‐10 antagonist, antineoplastic (glioblastoma multiforme), antineoplastic (bone cancer), catalase stimulant
4a‐Phorbol 12,13‐didecanoate	10.21	1.05	C_40_H_64_O_8_	672.9	Vanilloid agonist, protein kinase stimulant, antihelmintic, retinol dehydrogenase inhibitor, antipruritic, nonallergic, antiviral (rhinovirus)
Hydroquinone 1,4‐Benzenediol	10.85	2.87	C_6_H_6_O_2_	110.11	Carminative, nicotinic alpha 2 beta 2 receptor antagonist, vasoprotector, antieczematic, erythropoiesis stimulant
1‐Hexadecene	11.88	1.76	C_16_H_32_	224.42	Mucomembranous protector, fatty‐acyl‐CoA synthase inhibitor, ubiquinol‐cytochrome‐creductase inhibitor, gluconate 5‐dehydrogenase inhibitor, taurine dehydrogenase inhibitor
Methyl 3‐hydroxyl tetradecanoate Tetradecanoic acid, 3‐hydroxy‐, methyl ester	12.57	1.25	C_15_H_30_O_3_	258.4	Vasodilator, peripheral, membrane integrity agonist, intestinal, oxidizing agent, Preneoplastic conditions treatment
Salicylaldehyde hydrazone benzaldehyde, 2‐hydroxy‐, hydrazine	16.64	5.11	C_7_H_8_N_2_O	136.15	Cytoprotectant, antineoplastic (melanoma), nucleoside oxidase (H_2_O_2_‐forming), antithrombotic, alopecia treatment
α‐d‐Glucopyranose, 4‐*O*‐α‐d‐galactopyranosyl‐lactose, α‐	18.47	1.82	C_12_H_22_O_11_	342	Beta‐glucosidase inhibitor, licheninase inhibitor, osmotic diuretic, sucrose alpha‐glucosidase inhibitor, sweetener, anaphylatoxin receptor antagonist, xylose isomerase inhibitor
2‐Propenoic acid, 3‐(4‐fluorophenyl)‐, ethylester	18.68	1.23	C_11_H_11_FO_2_	219.21	Antipruritic, allergic, anti‐secretoric, antidiabetic, dermatologic, fibrinolytic, anti‐inflammatory, anti‐asthmatic, anti‐anginal
Î‐*N*‐Formyl‐l‐lysine	23.99	8.99	C_7_H_14_N_2_O_3_	174.2	Limulus clotting factor B inhibitor, Pseudolysin inhibitor, aspartate‐phenylpyruvate transaminase inhibitor, fibrolase inhibitor
Pentadecanoic acid, 13‐methyl‐, methyl ester	26.10	3.00	C_17_H_34_O_2_	270.5	Mucositis treatment, yeast ribonuclease inhibitor, methylum belliferyl‐acetate deacetylase inhibitor, vasodilator, peripheral, anti‐seborrheic
l‐(+)‐Ascorbicacid2,6‐dihexadecanoate	27.31	2.25	C_38_H_68_O_8_	652.9	Shikimate O‐hydroxycinnamoyl transferase inhibitor, kidney function stimulant, anti‐seborrheic, myeloblastin inhibitor, histidine *N*‐acetyl transferase inhibitor, platelet aggregation stimulant, carminative
4‐Cyclohepta‐2,4,6‐trienyl‐benzoicacid	30.54	0.96	C_14_H_12_O_2_	212.24	Mucomembranous protector, acylcarnitine hydrolase inhibitor, electron‐transferring‐flavoprotein dehydrogenase inhibitor, Pterindeaminase inhibitor
Octadecanoicacid,9,10‐dichloro‐, methyl ester	31.13	1.32	C_19_H_36_Cl_2_O_2_	367.4	Anti‐eczematic, pediculicide, mucositis treatment, preneoplastic conditions treatment, antimutagenic
5,6‐Dimethoxyphthal aldehydicacid	33.04	3.64	C_10_H_10_O_5_	210.8	Membrane permeability enhancer, aspartyl transferase inhibitor, pro‐opiomelanocortin converting enzyme inhibitor, fibrinolytic, anti‐seborrheic, antineoplastic
Hexadecanoicacid,1‐(2aminoethoxy) hydroxyphosphinyl]oxy]methyl]‐1,2‐ethanediylester	39.59	1.40	C_37_H_74_NO_8_P	692	Macrophage colony stimulating factor agonist, choline‐phosphate cytidylyl transferase inhibitor, analeptic, antithrombotic, respiratory analeptic, aspulvinone dimethylallyl transferase inhibitor
4‐Normethyl‐9,19 cyclolanostan‐7‐one,3 acetoxy‐	40.62	2.01	C_31_H_50_O_3_	470.7	Antifungal, prostate disorders treatment, immunosuppressant, antibacterial, anti‐ulcerative
l‐(+)‐Ascorbicacid2,6‐dihexadecanoate	27.31	2.25	C_38_H_68_O_8_	652.9	Shikimate O‐hydroxycinnamoyl transferase inhibitor, kidney function stimulant, anti‐seborrheic, myeloblast ininhibitor, histidine *N*‐acetyl transferase inhibitor, platelet aggregation stimulant, carminative
4‐Cyclohepta‐2,4,6‐trienyl‐benzoicacid	30.54	0.96	C_14_H_12_O_2_	212.24	Mucomembranous protector, acylcarnitine hydrolase inhibitor, electron‐transferring‐flavoprotein dehydrogenase inhibitor, Pterindeaminase inhibitor
Octadecanoicacid,9,10‐dichloro‐, methyl ester	31.13	1.32	C_19_H_36_Cl_2_O_2_	367.4	Anti‐eczematic, pediculicide, mucositis treatment, Preneoplastic conditions treatment, antimutagenic
Cyclodecasiloxane, eicosamethyl‐	32.22	1.66	C_20_H_60_O_10_Si_10_	741.5	Polyneuridine‐aldehyde esterase inhibitor, phobic disorders treatment, chloride peroxidase inhibitor, acetyl esterase inhibitor, feruloyl esterase inhibitor

Out of 27 compounds identified from SiEE, 15 compounds followed the Lipinski's five rule, namely, molecular weight, lipophilicity (LogP), number of hydrogen bond donors and acceptors and molar refractivity [43] and other 12 identified compounds do not meet the Lipinski's rule. Among the 27 identified compounds, 15 compounds physicochemical properties confirmed their suitability for oral drug.

## 4 ADME

**FIGURE 2:**
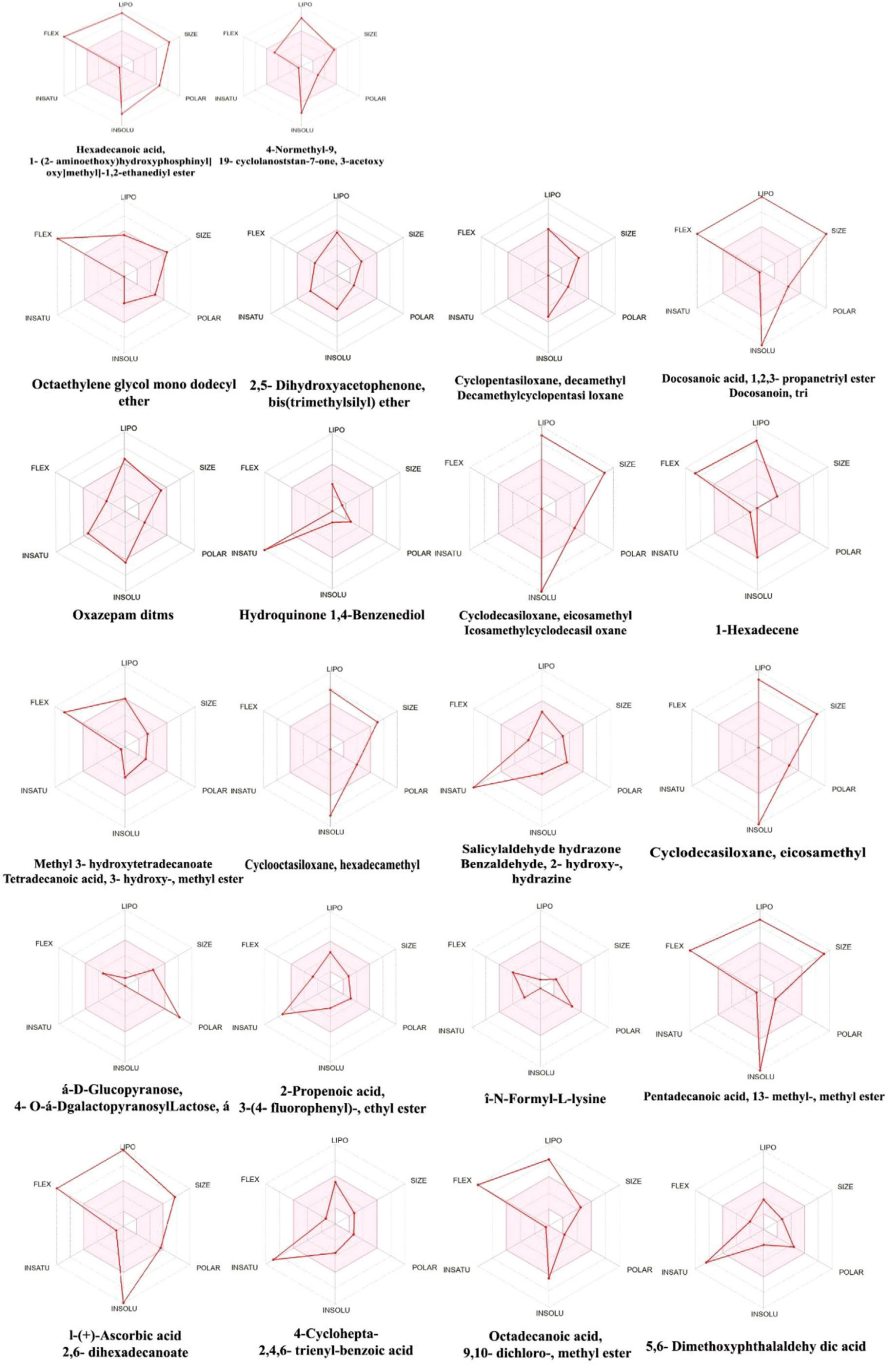
Swiss ADME bioavailability radar of Identified bioactive compounds of *Selaginella inaequalifolia* ethanolic extracts (where the pink areas represent each property, namely, lipophilicity, molecular weight, solubility, and flexibility).

The physicochemical and pharmacokinetic profiling of the compound library revealed a diverse range of molecular characteristics and drug‐like behaviors. The physicochemical properties have shown balanced molecular weight distribution, moderate hydrogen bonding capacity (HBD/HBA), and topological polar surface area (TPSA) values, with most compounds exhibiting acceptable numbers of rotatable bonds. Lipophilicity analysis (Consensus LogP) indicated that 59.1% of compounds fell within the optimal drug‐like range (LogP 1–5), though notable outliers such as docosanoic acid triglyceride (LogP 21.43) reflected algorithmic variability. Water solubility classification placed compounds into six solubility categories, identifying highly soluble molecules like *N*‐formyl‐l‐lysine (28,400 mg/mL) and α‐d‐glucopyranose, 4‐*O*‐α‐d‐galactopyranosyl lactose, α (696.0 mg/mL), as well as essentially insoluble ones like docosanoic acid triglyceride (8.56 × 10^−32^ mg/mL). Pharmacokinetic predictions showed 54.5% of compounds with high gastrointestinal (GI) absorption and 45.5% capable of crossing the blood–brain barrier, with bioavailability scores ranging from 0.17 to 0.85 of which the highest score is observed for 4‐cyclohepta‐2,4,6‐trienyl‐benzoic acid (0.85). Drug‐likeness analysis across five rule sets (Lipinski, Ghose, Veber, Egan, and Muegge) are categorized into three tiers: Tier 1 (optimal, 31.8% with 0–1 violations), Tier 2 (moderate, 31.8% with 2–4 violations), and Tier 3 (poor, 36.4% with ≥5 violations). The integrated assessment identified 4‐cyclohepta‐2,4,6‐trienyl‐benzoic acid and 2,5‐dihydroxyacetophenone bis(trimethylsilyl) ether as lead candidates for further development (Tables 2, 3, 4, 5, 6; Figure 2). Among them, nine compounds fully met all the criteria, including 2,5‐dihydroxyacetophenone, cyclopentasiloxane, Oxazepam ditms, hydroquinone, methyl 3‐hydroxytetradecanoate, salicylaldehyde hydrazone, î‐*N*‐formyl‐l‐lysine, pentadecanoic acid, and 4‐cyclohepta‐2,4,6‐trienyl‐benzoic acid. These compounds showed good potential as drug‐like molecules. On the other hand, three compounds—cyclodecasiloxane, cyclooctasiloxane, and α‐d‐glucopyranose failed to meet two or more rules of Lipinski, mostly due to their size and high number of hydrogen bond donors or acceptors, which may reduce their chances of being absorbed well in the body. A few other compounds like 1‐hexadecene, 2‐propenoic acid (ethyl ester), and 5,6‐dimethoxyphthalaldehydic acid had one or two rule violations, meaning they may still have potential but might face some issues with absorption or bioavailability. Overall, most of the compounds studied show promise for further drug development, especially those met Lipinski's criteria (Tables 2, 3, 4, 5, 6; Figure 2).

**TABLE 2:** Physicochemical properties of the identified compounds of *Selaginella inaequalifolia* ethanolic extracts.

Compounds	MW	HA	AHA	Fraction Csp3	RB	HBA	HBD	MR	TPSA
Octaethyleneglycolmonododecyl ether	538.75	37	0	1	34	9	1	146.55	94.07
2,5‐Dihydroxyacetophenone, bis(trimethylsilyl) ether	296.51	19	6	0.5	5	3	0	84.58	35.53
Cyclopentasiloxane, decamethylDecamethylcyclopentasiloxane	370.77	20	0	1	0	5	0	92.84	46.15
Docosanoic acid, 1,2,3‐propanetriyl ester docosanoin, tri	1059.80	75	0	0.96	68	6	0	337.65	78.90
Oxazepam ditms	431.08	28	12	0.33	4	3	0	128.81	41.9
Hydroquinone 1,4‐benzenediol	110.11	8	6	0	0	2	2	30.49	40.46
Cyclodecasiloxane, eicosamethylIcosamethylcyclodecasioxane	741.54	40	0	1	0	10	0	185.67	92.3
1‐Hexadecene	224.43	16	0	0.88	13	0	0	78.55	0
Methyl 3‐hydroxytetradecanoateTetradecanoic acid, 3‐hydroxy‐, methyl ester	258.4	18	0	0.93	13	3	1	76.67	46.53
Cyclooctasiloxane, hexadecamethyl	593.23	32	0	1	0	8	0	148.54	73.84
Salicylaldehyde hydrazone benzaldehyde, 2‐hydroxy‐, hydrazine	240.26	18	12	0	3	4	2	72.12	65.18
Cyclodecasiloxane, eicosamethyl	741.54	40	0	1	0	10	0	185.67	92.3
α‐d‐Glucopyranose, 4‐*O*‐α‐d‐galactopyranosyl Lactose, α	342.3	23	0	1	5	11	8	68.16	189.53
2‐Propenoic acid, 3‐(4‐fluorophenyl)‐, ethyl ester	219.21	16	6	0.17	4	4	0	56.75	50.09
î‐*N*‐Formyl‐l‐lysine	174.2	12	0	0.71	6	4	3	43.24	92.42
Pentadecanoic acid, 13‐methyl‐, methyl ester	778.15	47	0	0.94	15	31	0	82.32	26.3
l‐(+)‐Ascorbic acid 2,6‐dihexadecanoate	652.94	46	0	0.87	34	8	2	188.78	119.36
4‐Cyclohepta‐2,4,6‐trienyl‐benzoic acid	212.24	16	6	0.07	2	2	1	63.67	37.3
Octadecanoic acid, 9,10‐dichloro‐, methyl ester	367.39	23	0	0.95	1	2	0	104.32	26.30
5,6‐Dimethoxyphthalaldehydic acid	210.18	15	6	0.1	3	5	3	53.65	86.99
Hexadecanoic acid, 1‐(2‐aminoethoxy)hydroxyphosphinyl]oxy]methyl]‐1,2‐ethanediyl ester	691.96	47	0	0.95	39	9	2	197.25	144.19
4‐Normethyl‐9,19‐cyclolanoststan‐7‐one, 3‐acetoxy	470.73	34	0	0.94	7	3	0	141.01	43.37

Abbreviations: AHA, number aromatic heavy atoms; HA, number heavy atoms; HBA, number hydrogen bond acceptor; HBD, number hydrogen bound donor; MR, molar refractivity (m^3^/mol); MW, molecular weight (g/mol); RB, number rotatable bonds; TPSA, topology polar surface area (Å^2^).

**TABLE 3:** Lipophilicity characteristics of the identified compounds of *Selaginella inaequalifolia* ethanolic extracts.

Compounds	iLOGP	XLOGP3	WLOGP	MLOGP	SILICOS‐IT	Consensus Log P
Octaethylene glycol mono dodecyl ether	7.59	4.06	4.03	0.41	7.57	4.73
2,5‐Dihydroxyacetophenone, bis(trimethylsilyl) ether	3.84	4.39	4.32	2.24	0.84	3.13
Cyclopentasiloxane, decamethylDecamethylcyclopentasiloxane	4.4	5.03	3.59	−1.04	−4.09	1.58
Docosanoic acid, 1,2,3‐propanetriyl ester Docosanoin, tri	13.64	31.68	23.45	11.45	26.93	21.43
Oxazepam ditms	4.18	6.05	4.77	3.77	2.33	4.22
Hydroquinone 1,4‐benzenediol	0.92	0.59	1.1	0.79	0.94	0.87
Cyclodecasiloxane, eicosamethylIcosamethylcyclodecasioxane	6.55	10.06	7.18	−2.43	−9.11	2.45
1‐Hexadecene	4.61	8.95	6.26	6.29	6.2	6.46
Methyl 3‐hydroxytetradecanoateTetradecanoic acid, 3‐hydroxy‐, methyl ester	3.88	5.02	3.83	3.06	4.23	4
Cyclooctasiloxane, hexadecamethyl	5.85	8.05	5.75	−1.83	−7.1	2.14
Salicylaldehyde hydrazone Benzaldehyde, 2‐hydroxy‐, hydrazine	1.87	2.59	2.55	1.87	3.23	2.42
Cyclodecasiloxane, eicosamethyl	6.55	10.06	7.18	−2.43	−9.11	2.45
α‐d‐Glucopyranose, 4‐*O*‐α‐D–galactopyranosylLactose, α	0.85	−3.7	−5.4	−4.37	−3.86	−3.29
2‐Propenoic acid, 3‐(4‐fluorophenyl)‐, ethyl ester	2.18	2.53	2.61	2.21	2.76	2.46
î‐*N*‐Formyl‐l‐lysine	0.61	−3.57	−0.69	−2.85	−0.62	−1.42
Pentadecanoic acid, 13‐methyl‐, methyl ester	6.25	9.96	21.17	7.3	10.79	11.09
l‐(+)‐Ascorbic acid 2,6‐dihexadecanoate	8.16	14.06	10.09	4.64	11.49	9.69
4‐Cyclohepta‐2,4,6‐trienyl‐benzoic acid	1.71	3.64	3.15	3.02	2.04	2.71
Octadecanoic acid, 9,10‐dichloro‐, methyl ester	4.87	8.69	6.86	5.36	7.49	6.65
5,6‐Dimethoxyphthalaldehydic acid	1.6	1.13	1.1	0.44	0.81	1.01
Hexadecanoic acid, 1‐(2‐aminoethoxy)hydroxyphosphinyl]oxy]methyl]‐1,2‐ethanediyl ester	7.78	10.26	10.50	4.98	11.30	8.98
4‐Normethyl‐9,19‐cyclolanoststan‐7‐one, 3‐acetoxy	4.89	8.78	7.61	6.01	7.38	6.94

**TABLE 4:** Water solubility characteristics of the identified compounds of *Selaginella inaequalifolia* ethanolic extracts.

Compounds	Log S	Solubility		Class
		mol/L	mg/mL	
Octaethyleneglycolmonododecyl ether	−3.49	1.82e−06	9.81e−04	Moderately soluble
2,5‐Dihydroxyacetophenone, bis(trimethylsilyl) ether	−4.35	1.40E−05	4.16E−03	Moderately soluble
Cyclopentasiloxane, decamethylDecamethylcyclopentasiloxane	−5.31	1.82E−06	6.75E−04	Soluble
Docosanoic acid, 1,2,3‐propanetriyl ester Docosanoin, tri	−34.09	8.08E−35	8.56E−32	Insoluble
Oxazepam ditms	−6.38	1.95E−07	8.42E−0e−5	Poorly soluble
Hydroquinone 1,4‐Benzenediol	−1.45	9.70E−02	1.07E+01	Soluble
Cyclodecasiloxane, eicosamethylIcosamethylcyclodecasioxane	−10.78	1.18E−12	8.74E−10	Moderately soluble
1‐Hexadecene	−6.01	1.45E−09	3.25E−07	Moderately soluble
Methyl 3‐hydroxytetradecanoateTetradecanoic acid, 3‐hydroxy‐, methyl ester	−3.75	1.83E−06	4.73E−04	Moderately soluble
Cyclooctasiloxane, hexadecamethyl	−8.59	3.50E−10	2.08E−07	Moderately soluble
Salicylaldehyde hydrazone Benzaldehyde, 2‐hydroxy‐, hydrazine	−3.26	2.47E−04	5.93E−02	Soluble
Cyclodecasiloxane, eicosamethyl	−10.78	1.18E−12	8.74E−10	Insoluble
α‐d‐Glucopyranose, 4‐*O*‐α‐d‐galactopyranosylLactose, α	0.7	2.03e+00	6.96E+02	Soluble
2‐Propenoic acid, 3‐(4‐fluorophenyl)‐, ethyl ester	−2.81	5.91E−04	1.30E−01	Soluble
î‐*N*‐Formyl‐l‐lysine	1.73	1.63e+02	2.84E+04	Soluble
Pentadecanoic acid, 13‐methyl‐, methyl ester	−9.95	3.64E−11	2.83E−08	Poorly soluble
l‐(+)‐Ascorbic acid 2,6‐dihexadecanoate	−10.50	2.25E−17	1.47E−14	Insoluble
4‐Cyclohepta‐2,4,6‐trienyl‐benzoic acid	−3.59	7.73E−05	1.64E−02	Soluble
Octadecanoic acid, 9,10‐dichloro‐, methyl ester	−6.47	7.75E−10	2.78E−07	Poorly soluble
5,6‐Dimethoxyphthalaldehydic acid	−1.95	2.81E−03	5.92E−01	Soluble
Hexadecanoic acid, 1‐(2‐aminoethoxy)hydroxyphosphinyl]oxy]methyl]‐1,2‐ethanediyl ester	−8.02	5.94E−14	4.11E−11	Insoluble
4‐Normethyl‐9,19‐cyclolanoststan‐7‐one, 3‐acetoxy	−7.83	2.67E−07	1.26E−10	Poorly soluble

*Note*: Insoluble < −10 < poorly soluble (PS) < −6 < moderately soluble (MS) < −4 < soluble < −2 < very soluble (VS) < 0 < Highly Soluble (HS).

**TABLE 5:** Pharmacokinetics parameters and bioavailability of the identified compounds of *Selaginella inaequalifolia* ethanolic extracts.

Compounds	GI absorption	BBB permeant	Bioavailability score
Octaethyleneglycolmonododecyl ether	High	**No**	0.55
2,5‐Dihydroxyacetophenone, bis(trimethylsilyl) ether	High	Yes	0.55
Cyclopentasiloxane, decamethylDecamethylcyclopentasiloxane	High	Yes	0.55
Docosanoic acid, 1,2,3‐propanetriyl ester Docosanoin, tri	Low	No	0.17
Oxazepam ditms	High	Yes	0.55
Hydroquinone 1,4‐Benzenediol	High	Yes	0.55
Cyclodecasiloxane, eicosamethylIcosamethylcyclodecasioxane	Low	No	0.55
1‐Hexadecene	Low	No	0.55
Methyl 3‐hydroxytetradecanoateTetradecanoic acid, 3‐hydroxy‐, methyl ester	High	Yes	0.55
Cyclooctasiloxane, hexadecamethyl	High	No	0.55
Salicylaldehyde hydrazone Benzaldehyde, 2‐hydroxy‐, hydrazine	High	Yes	0.55
Cyclodecasiloxane, eicosamethyl	Low	No	0.55
α‐d‐Glucopyranose, 4‐*O*‐α‐D–galactopyranosylLactose, α	Low	No	0.17
2‐Propenoic acid, 3‐(4‐fluorophenyl)‐, ethyl ester	High	Yes	0.55
î‐*N*‐Formyl‐l‐lysine	High	No	0.55
Pentadecanoic acid, 13‐methyl‐, methyl ester	Low	No	0.17
l‐(+)‐Ascorbic acid 2,6‐dihexadecanoate	Low	No	0.56
4‐Cyclohepta‐2,4,6‐trienyl‐benzoic acid	High	Yes	0.85
Octadecanoic acid, 9,10‐dichloro‐, methyl ester	Low	No	0.55
5,6‐Dimethoxyphthalaldehydic acid	High	No	0.56
Hexadecanoic acid, 1‐(2‐aminoethoxy)hydroxyphosphinyl]oxy]methyl]‐1,2‐ethanediyl ester	Low	No	0.17
4‐Normethyl‐9,19‐cyclolanoststan‐7‐one, 3‐acetoxy	Low	No	0.55

Abbreviations: BBB permeant, blood–brain barrier permeation; GI absorption, gastrointestinal absorption.

**TABLE 6:** Drug‐likeness rules score of the identified compounds of *Selaginella inaequalifolia* ethanolic extracts.

Compounds	Lipinski violations	Ghose violations	Veber violations	Egan violations	Muegge violations
Octaethyleneglycolmonododecyl ether	1	3	1	1	1
2,5‐Dihydroxyacetophenone, bis(trimethylsilyl) ether	0	0	0	0	0
Cyclopentasiloxane, decamethylDecamethylcyclopentasiloxane	0	0	0	0	1
Docosanoic acid, 1,2,3‐propanetriyl ester Docosanoin, tri	2	4	1	1	3
Oxazepam ditms	0	0	0	0	1
Hydroquinone 1,4‐Benzenediol	0	3	0	0	1
Cyclodecasiloxane, eicosamethylIcosamethylcyclodecasioxane	1	4	0	1	2
1‐Hexadecene	1	1	1	1	2
Methyl 3‐Hydroxytetradecanoatetetradecanoic acid, 3‐hydroxy‐, methyl ester	0	0	1	0	1
Cyclooctasiloxane, hexadecamethyl	1	4	0	0	1
Salicylaldehyde hydrazone Benzaldehyde, 2‐hydroxy‐, hydrazine	0	0	0	0	0
Cyclodecasiloxane, eicosamethyl	1	4	1	1	2
α‐d‐Glucopyranose, 4‐*O*‐α‐d‐galactopyranosylLactose, α	2	1	1	1	4
2‐Propenoic acid, 3‐(4‐fluorophenyl)‐, ethyl ester	0	0	0	0	0
î‐*N*‐Formyl‐l‐lysine	0	1	0	0	2
Pentadecanoic acid, 13‐methyl‐, methyl ester	2	2	1	1	3
l‐(+)‐Ascorbic acid 2,6‐dihexadecanoate	2	4	1	1	3
4‐Cyclohepta‐2,4,6‐trienyl‐benzoic acid	0	0	0	0	0
Octadecanoic acid, 9,10‐dichloro‐, methyl ester	1	1	1	1	2
5,6‐Dimethoxyphthalaldehydic acid	0	0	0	0	0
Hexadecanoic acid, 1‐(2‐aminoethoxy)hydroxyphosphinyl]oxy]methyl]‐1,2‐ethanediyl ester	2	4	2	2	3
4‐Normethyl‐9,19‐cyclolanoststan‐7‐one, 3‐acetoxy	1	3	1	1	1

*Note*: A—Molecular mass less than 500 Da; B—high lipophilicity (expressed as Log P less than 5); C—less than 5 hydrogen bond donors; D—less than 10 hydrogen bond acceptors; E—molar refractivity should be between 40 and 130; F—overall.

Figure 2 visuals interpretation of individual radar plots revealed three clear tiers of compound performance based on ADME parameters. Tier 1 (optimal candidates), represented by large and regular hexagons, included compounds such as octaethylene glycol mono dodecyl ether, 2,5‐dihydroxyacetophenone bis(trimethylsilyl) ether, cyclopentasiloxane decamethyl, hydroquinone (1,4‐benzenediol), and salicylaldehyde hydrazone, all shown polygons extending uniformly toward the outer edges, indicating strong and balanced ADME profiles across all six parameters. Tier 2 (moderate candidates), shown as medium‐sized polygons with slight indentations, comprised compounds like Oxazepam ditms, cyclooctasiloxane hexadecamethyl, methyl 3‐hydroxytetradecanoate, 4‐d‐glucopyranose derivatives, and 2‐propenoic acid, 3‐(4‐fluorophenyl)‐ethyl ester, each displaying good overall performance but with specific weaknesses such as reduced solubility or excessive lipophilicity. Tier 3 (poor candidates) featured small, irregular polygons with deep indentations, including docosanoic acid, 1,2,3‐propanetryl ester, 1‐hexadecene, cyclodecasiloxane eicosamethyl, pentadecanoic acid, 13‐methyl‐, methyl ester, 1‐(+)‐ascorbic acid 2,6‐dihexadecanoate, and octadecanoic acid, 9,10‐dichloro‐, methyl ester, all showing pronounced ADME limitations such as high molecular weight, low solubility, or poor bioavailability. Interestingly, 4‐cyclohepta‐2,4,6‐trienyl‐benzoic acid stood out within this group as an exception, forming one of the largest and most regular polygons, reflecting an excellent and well‐balanced ADME profile overall.

Figure 2 shows the selected bioactive compounds identified through GC–MS, showing their 2D chemical structures, molecular docking interactions, and pharmacokinetic profiles. The accompanying radar plots represent drug‐likeness parameters such as lipophilicity, molecular weight, hydrogen bonding, and solubility, indicating their compliance with pharmacokinetic criteria [53]. Overall, the figure summarizes the structural diversity, binding potential, and ADMET properties of the analyzed compounds.

## 5 STOP TOX

The STOP TOX toxicity analysis of the 22 compounds identified from *Si*EE assessed six major toxicity endpoints acute inhalation (ACI), acute oral (ACO), acute dermal (ACD), eye irritation and corrosion (EIC), skin sensitization (SS), and skin irritation and corrosion (SIC) to evaluate pharmaceutical safety and development potential. The results revealed a complex relationship between ADME characteristics and toxicity behavior, where several compounds with strong drug‐like profiles exhibited certain toxicity concerns, while some poorly bioavailable molecules showed minimal hazards. Among the Tier 1 ADME candidates, 4‐cyclohepta‐2,4,6‐trienyl‐benzoic acid emerged as the most promising compound, displaying nontoxic behavior across all endpoints except for mild eye irritation. 2,5‐Dihydroxyacetophenone bis(trimethylsilyl) ether also showed favorable toxicity results with only minor dermal sensitivity, and *N*‐formyl‐l‐lysine demonstrated an excellent safety profile with minimal irritation potential. In contrast, salicylaldehyde hydrazone and hydroquinone, despite good ADME properties, showed multiple toxicity alerts including oral, dermal, and eye irritation concerns, likely requiring further validation or formulation adjustments. 2‐Propenoic acid 3‐(4‐fluorophenyl) ethyl ester exhibited oral and inhalation toxicity, possibly due to reactive intermediates. Interestingly, some Tier 3 ADME compounds such as docosanoic acid triglyceride and 1‐hexadecene showed low toxicity but remain pharmaceutically unsuitable due to poor solubility and bioavailability. Molecular toxicity mapping further visualized these risks, where green areas indicated safe regions and red zones marked toxic structural features. Based on integrated ADME–toxicity profiling, 4‐cyclohepta‐2,4,6‐trienyl‐benzoic acid, 2,5‐dihydroxyacetophenone bis(trimethylsilyl) ether, and *N*‐formyl‐l‐lysine are identified as Priority 1 lead candidates; salicylaldehyde hydrazone and hydroquinone as Priority 2 (requiring further assessment); and compounds such as oxazepam ditms and other poorly soluble lipids as Priority 3 (deprioritized). Overall, these findings highlight that both favorable ADME and low toxicity are essential for drug development potential, with select compounds demonstrating strong promise for subsequent bioactivity screening and pharmacokinetic validation (Figure 3A, 3B, 3C; Table 7).

FIGURE 3(A–C) Toxic properties of the identified compounds of *Selaginella inaequalifolia* ethanolic extracts. Green areas show parts of the molecule that have lower toxicity (safe features), while red areas show the parts that increase toxicity (risky features). Darker colors mean a stronger effect, and lighter, fuzzy edges mean the model is less certain there. A sharp red dot highlights a specific atom or bond with a high toxicity risk.

**Figure:**
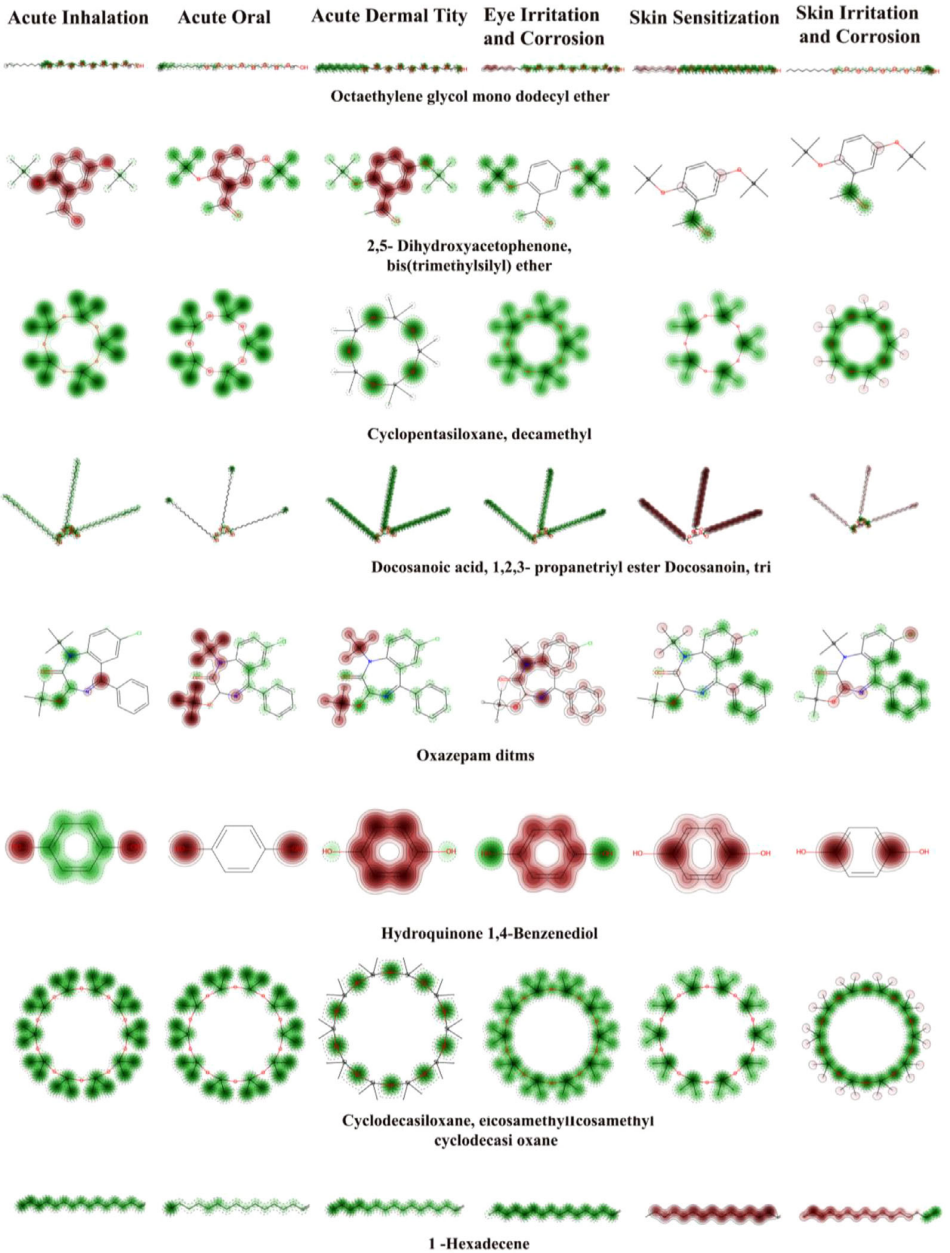

**Figure:**
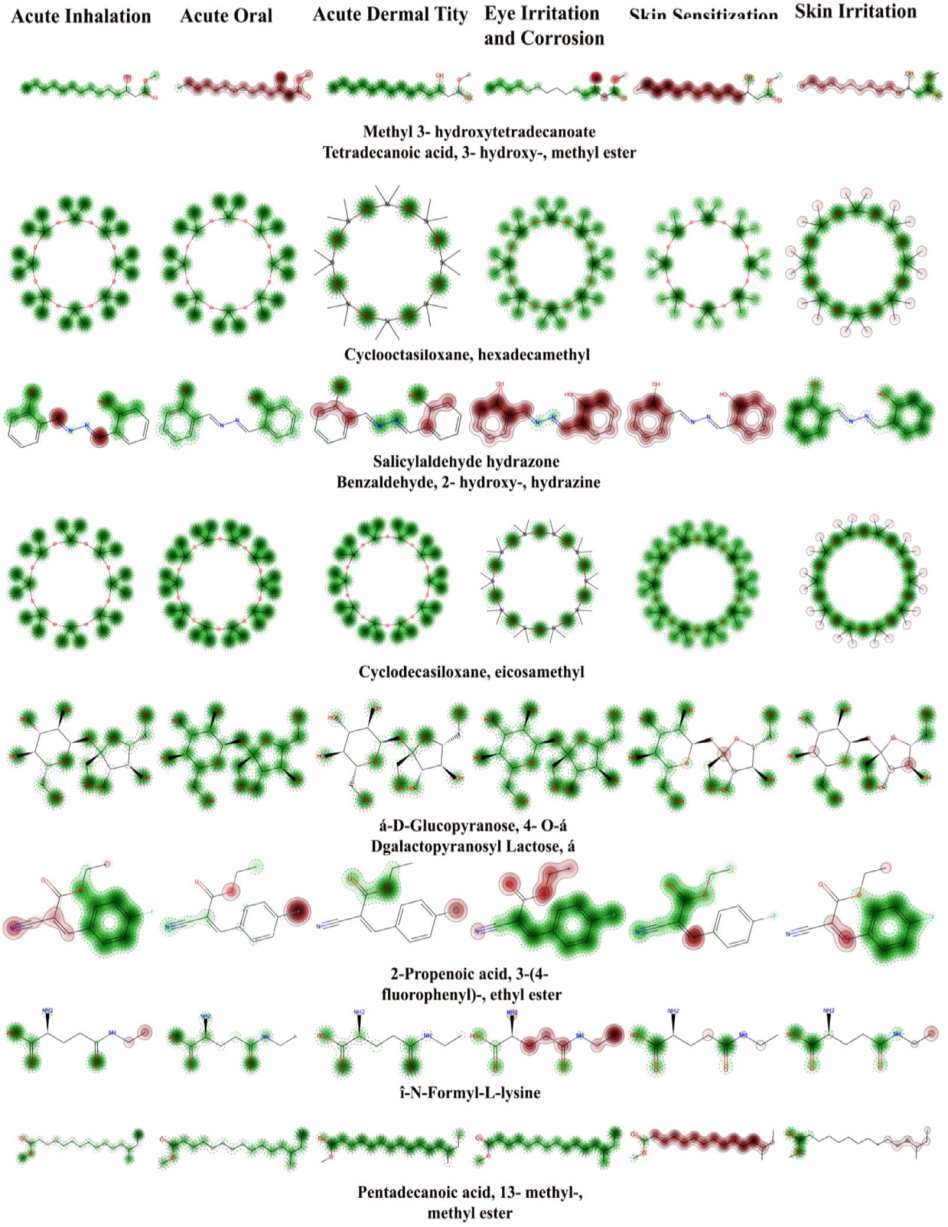

**Figure:**
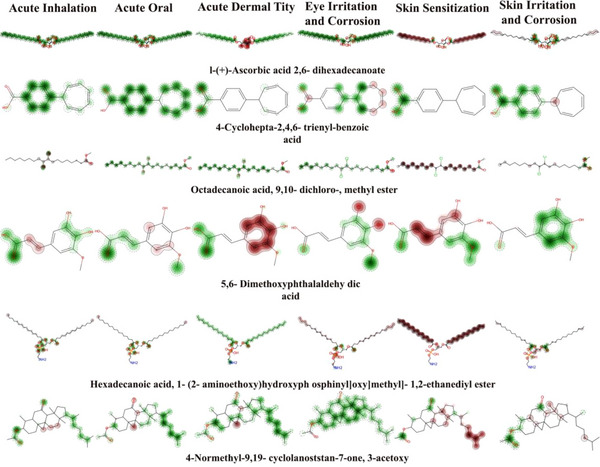

**TABLE 7:** Toxic properties of identified compounds of *Selaginella inaequalifolia* ethanolic extracts—STOP TOX prediction.

Compound name	ACI	ACO	ACD	EIC	SS	SIC
Octaethylene glycol mono dodecyl ether	NT	NT	NT	NT	NS	+
2,5‐Dihydroxyacetophenone, bis(trimethylsilyl) ether	NT	NT	T	NT	NS	−
Cyclopentasiloxane, decamethyl	NT	NT	NT	NT	NS	+
Docosanoic acid, 1,2,3‐propanetriyl ester Docosanoin, tri	NT	NT	NT	NT	S	−
Oxazepam ditms	T	T	NT	T	S	−
Hydroquinone 1,4‐Benzenediol	NT	T	T	T	S	+
Cyclodecasiloxane	NT	NT	NT	NT	NS	+
1‐Hexadecene	NT	NT	NT	NT	S	+
Methyl 3‐hydroxytetradecanoateTetradecanoic acid, 3‐hydroxy‐, methyl ester	NT	NT	NT	NT	S	+
Cyclooctasiloxane, hexadecamethyl	NT	NT	NT	NT	S	+
Salicylaldehyde hydrazone Benzaldehyde, 2‐hydroxy‐, hydrazine	T	NT	T	T	S	−
Cyclodecasiloxane, eicosamethyl	NT	NT	NT	NT	S	+
α‐d‐Glucopyranose, 4‐*O*‐α‐d‐galactopyranosyl Lactose, α	NT	NT	NT	NT	NS	−
2‐Propenoic acid, 3‐(4‐fluorophenyl)‐, ethyl ester	T	T	NT	NT	NS	−
î‐*N*‐Formyl‐l‐lysine	NT	NT	NT	T	NS	−
Pentadecanoic acid, 13‐methyl‐, methyl ester	NT	NT	NT	NT	S	−
l‐(+)‐Ascorbic acid 2,6‐dihexadecanoate	NT	NT	NT	NT	S	−
4‐Cyclohepta‐2,4,6‐trienyl‐benzoic acid	NT	NT	NT	T	NS	−
Octadecanoic acid, 9,10‐dichloro‐, methyl ester	T	NT	NT	NT	S	+
5,6‐Dimethoxyphthalaldehydic acid	NT	NT	NT	NT	S	−
Hexadecanoic acid, 1‐(2‐aminoethoxy)hydroxyph osphinyl]oxy]methyl]‐1,2‐ethanediyl ester	NT	NT	NT	T	S	−
4‐Normethyl‐9,19‐cyclolanoststan‐7‐one, 3‐acetoxy	NT	NT	NT	NT	NS	−

Abbreviations: ACD, acute dermal tity; ACI, acute inhalation; ACO, acute oral; EIC, eye irritation and corrosion; NS, non‐sensitizer; NT, nontoxic; S, sensitizer; SIC, skin irritation and corrosion; SS, skin sensitization; T, toxic.

### 5.1 Docking Analysis of Ligand Against CDK2 (PDB ID: 4GCJ)

**FIGURE 4:**
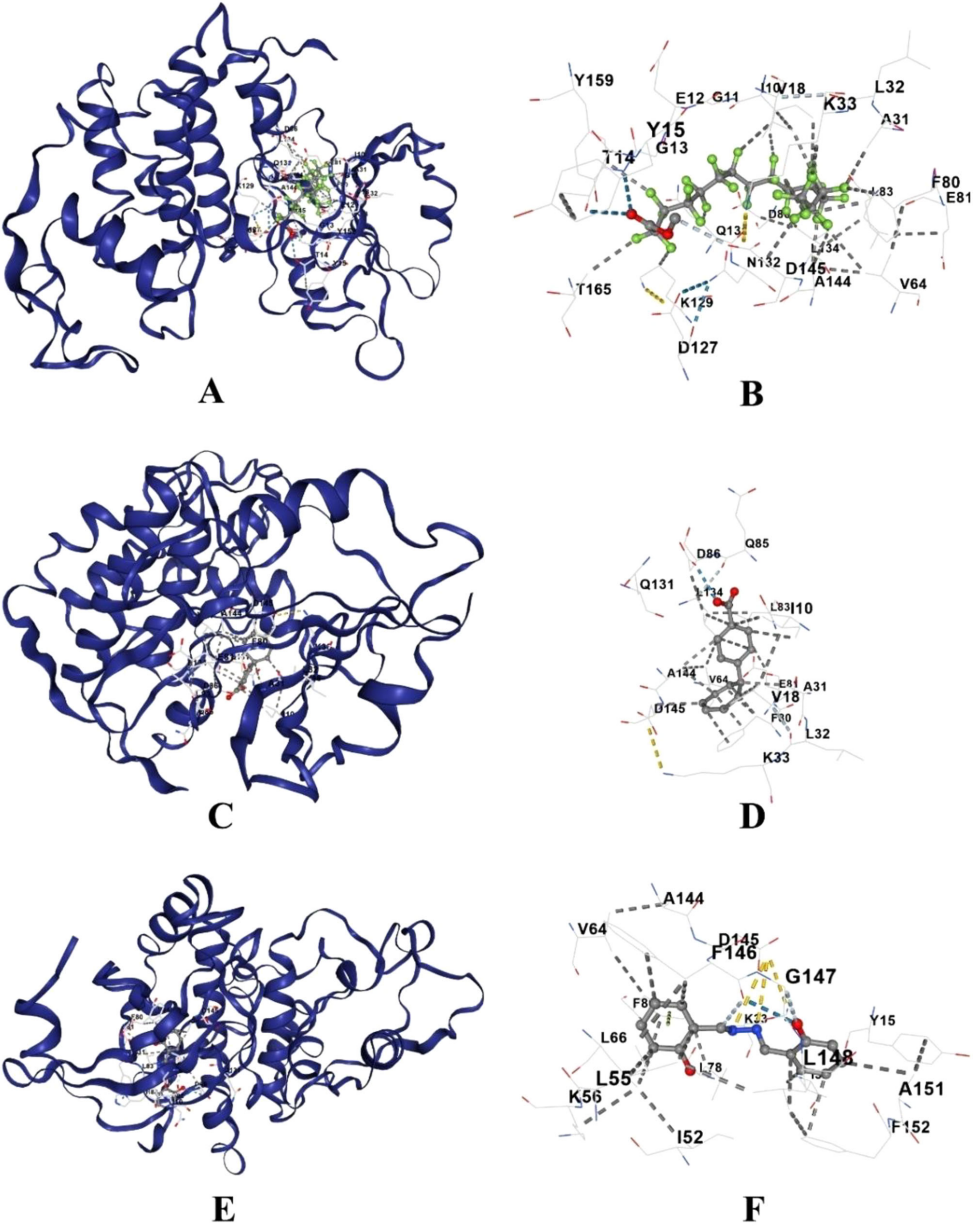
Molecular docking of identified compounds of *Si*EE against cyclin‐dependent kinase 2 (CDK2) (PDB ID: 4GCJ) (A, B) Pentadecanoic acid, 13‐methyl‐, methyl ester; (C, D) 4‐cyclohepta‐2,4,6‐trienyl‐benzoic acid, (E, F) salicylaldehyde hydrazone benzaldehyde, 2‐hydroxy‐, hydrazine.

A high binding affinity is observed between Pentadecanoic acid, 13‐methyl‐, methyl ester; 4‐cyclohepta‐2,4,6‐trienyl‐benzoic acid, salicylaldehyde hydrazone, 2‐propenoic acid, 3‐(4‐fluorophenyl)‐, ethyl ester; and Oxazepam ditmsa gainst CDK2. Among these, pentadecanoic acid, 13‐methyl‐, methyl ester exhibited the strongest binding affinity with a Vina score of −11.0 kcal/mol, followed by 4‐cyclohepta‐2,4,6‐trienyl‐benzoic acid (−8.9 kcal/mol) and salicylaldehyde hydrazone (−8.2 kcal/mol). Oxazepam ditms also showed a significant interaction with a Vina score of −7.0 kcal/mol, forming strong contacts with key residues in the active site (Table 8). The identified compounds with Vina scores of −5 and above are included in the Table 8. The most common and prominent interacting residues among these compounds included GLU8, GLY13, THR14, TYR15, ASP127, PHE146, GLY147, LEU148, ARG150, and PHE152, which play crucial roles in kinase activity regulation (Figure 4; Table 8). These findings suggested that these compounds may serve as potential CDK2 inhibitors (Table 8).

**TABLE 8:** In silico docking of identified compounds from *Selaginella inaequalifolia* ethanolic extracts against cyclin‐dependent kinase 2 (CDK2)—4GCJ and Serine/threonine‐protein kinase mTOR—4JSX.

Drug‐likeness ligands	Cavities	Vina score	Contact residue
Cyclin‐dependent kinase 2 (CDK2)—4GCJ			
2,5‐Dihydroxyacetophenone, bis(trimethylsilyl) ether	176	−5.5	**Chain A**: GLY13 THR14 TYR15 GLY16 LYS33 ARG126 ASP127 LYS129 ASP145 PHE146 GLY147 LEU148 ALA149 ARG150 ALA151 PHE152 GLU162 THR165 ARG169 TYR180
Cyclopentasiloxane, decamethylDecamethylcyclopentasiloxane	176	−4.7	**Chain A**: THR14 TYR15 HIS125 ARG126 ASP127 LYS129 GLY147 LEU148 ALA149 ARG150 ALA151 GLU162 THR165 ARG169 ILE173 TYR180 GLU208
Oxazepam ditms	176	−7.0	**Chain A**: GLY13 THR14 TYR15 GLY16 LYS33 HIS125 ARG126 ASP127 LEU128 LYS129 ASP145 PHE146 GLY147 LEU148 ALA149 ARG150 ALA151 GLU162 THR165 TYR168 ARG169 ILE173 LYS178 TYR180 SER188
Hydroquinone 1,4‐Benzenediol	3275	−5.4	**Chain A**: ILE10 TYR15 VAL18 ALA31 LYS33 ILE35 LEU55 LYS56 ILE63 VAL64 LYS65 LEU66 LEU78 PHE80 GLU81 PHE82 LEU83 HIS84 GLN85 ASP86 GLN131 ASN132 LEU134 LEU143 ALA144 ASP145 PHE146 GLY147 LEU148 ALA151 PHE152 GLY153
Cyclodecasiloxane	176	−5.8	**Chain A**: THR14 TYR15 ARG126 LEU148 ALA149 ARG150 ALA151 GLU162 THR165 ARG169 ILE173 LEU174 GLY176 CYS177 LYS178 TYR180 GLU208 ASP235
1‐Hexadecene	3275	−6.5	**Chain A**: GLU8 LYS9 ILE10 GLY11 GLU12 GLY13 TYR15 VAL18 LYS20 ALA31 LYS33 ILE35 ILE52 LEU55 LYS56 LEU58 ILE63 VAL64 LEU66 LEU78 PHE80 GLU81 PHE82 LEU83 HIS84 GLN85 ASP86 LYS89 ASP127 LYS129 GLN131 ASN132 LEU134 ALA144 ASP145 PHE146 GLY147 LEU148 ARG150 ALA151 PHE152 TYR159 THR165
Methyl 3‐hydroxytetradecanoateTetradecanoic acid, 3‐hydroxy‐, methyl ester	3275	−6.3	**Chain A**: GLU8 ILE10 GLY11 GLU12 GLY13 THR14 TYR15 GLY16 VAL18 LYS20 ALA31 LYS33 ILE35 ILE52 LEU55 LYS56 LEU58 ILE63 VAL64 LEU66 LEU78 PHE80 GLU81 PHE82 LEU83 HIS84 GLN85 ASP86 LYS89 ASP127 LYS129 GLN131 ASN132 LEU134 ALA144 ASP145 PHE146 GLY147 LEU148 ARG150 ALA151 PHE152 TYR159 THR165
Salicylaldehyde hydrazone Benzaldehyde, 2‐hydroxy‐, hydrazine	3275	−8.2	**Chain A**: GLU8 ILE10 GLU12 GLY13 THR14 TYR15 GLY16 VAL18 ALA31 LYS33 ILE35 ILE52 LEU55 LYS56 VAL64 LEU66 LEU78 PHE80 GLU81 PHE82 LEU83 HIS84 GLN85 ASP86 LYS89 ASP127 LYS129 GLN131 ASN132 LEU134 ALA144 ASP145 PHE146 GLY147 LEU148 ALA151 PHE152 TYR159 THR165
α‐d‐Glucopyranose, 4‐*O*‐α‐d‐galactopyranosylLactose, α	176	−6.3	**Chain A**: GLY13 THR14 TYR15 GLY16 LYS33 HIS125 ARG126 ASP127 LYS129 ASN132 ASP145 PHE146 GLY147 LEU148 ALA149 ARG150 ALA151 PHE152 TYR159 GLU162 THR165 LEU166 ARG169 TYR180
2‐Propenoic acid, 3‐(4‐fluorophenyl)‐, ethyl ester	3275	−7.1	**Chain A**: GLU8 ILE10 GLY11 GLU12 GLY13 THR14 TYR15 GLY16 VAL18 ALA31 LYS33 ILE35 ILE52 VAL64 LEU78 PHE80 GLU81 PHE82 LEU83 HIS84 GLN85 ASP86 ARG126 ASP127 LYS129 GLN131 ASN132 LEU134 ALA144 ASP145 PHE146 GLY147 LEU148 ALA151 PHE152 TYR159 THR165
î‐*N*‐Formyl‐l‐lysine	176	−5.7	**Chain A**: GLU12 GLY13 THR14 TYR15 LYS33 HIS125 ARG126 ASP127 LEU128 LYS129 GLN131 ASN132 ASP145 PHE146 GLY147 LEU148 ARG150 ALA151 PHE152 TYR159 GLU162 VAL164 THR165 LEU166 TYR168 ARG169 ALA170 ILE173 TYR180 VAL184 SER188
Pentadecanoic acid, 13‐methyl‐, methyl ester	3275	−11.0	**Chain A**: GLU8 LYS9 ILE10 GLY11 GLU12 GLY13 THR14 TYR15 GLY16 VAL18 TYR19 ALA31 LEU32 LYS33 ILE35 VAL64 LEU78 PHE80 GLU81 PHE82 LEU83 HIS84 GLN85 ASP86 LYS88 LYS89 ASP92 ASP127 LYS129 GLN131 ASN132 LEU134 ALA144 ASP145 PHE146 GLY147 LEU148 ARG150 ALA151 PHE152 TYR159 THR165
4‐Cyclohepta‐2,4,6‐trienyl‐benzoic acid	3275	−8.9	**Chain A**: GLU8 LYS9 ILE10 GLY11 GLU12 GLY13 THR14 TYR15 GLY16 VAL17 VAL18 ALA31 LEU32 LYS33 ILE35 ILE52 VAL64 LEU78 PHE80 GLU81 PHE82 LEU83 HIS84 GLN85 ASP86 LYS89 ASP127 LYS129 GLN131 ASN132 LEU134 ALA144 ASP145 PHE146 GLY147 LEU148 ARG150 ALA151 PHE152 TYR159
5,6‐Dimethoxyphthalaldehydic acid	3275	−6.6	**Chain A**: ILE10 GLU12 GLY13 VAL18 ALA31 LEU32 LYS33 VAL64 PHE80 GLU81 PHE82 LEU83 HIS84 GLN85 ASP86 GLN131 ASN132 LEU134 ALA144 ASP145 PHE146
Serine/threonine‐protein kinase mTOR—4JSX			
2,5‐Dihydroxyacetophenone, bis(trimethylsilyl) ether	4675	−6.9	**Chain B**: THR2279 MET2281 GLN2282 **Chain D**: ASN46 ALA47 LEU48 GLU49 ALA89 SER90 VAL91 GLY92 PHE93 GLU95 ASN132 CYS133 VAL134 CYS135 LEU136 PRO138 GLN140 THR174 SER175 ALA176 HIS177 ILE178 ASP179 PRO180 LEU224 GLN225 CYS226 ARG227 TRP274 GLY275 CYS276 VAL316 CYS317 LEU318
Cyclopentasiloxane, decamethylDecamethylcyclopentasiloxane	4675	−5.9	**Chain D**: GLU49 VAL50 THR51 PRO52 VAL91 GLY92 PHE93 GLU95 CYS135 LEU136 HIS137 PRO138 GLN140 HIS177 ILE178 ASP179 PRO180 ARG227 PHE228 SER229 PRO230 ALA277 PHE278 SER279 GLY280 ALA319 PHE320 ASN321 ASP322 SER323
Oxazepam ditms	3178	−7.4	**Chain C**: GLU49 VAL50 THR51 PRO52 VAL91 GLY92 PHE93 HIS94 GLU95 VAL134 CYS135 LEU136 HIS137 PRO138 GLN140 ALA176 HIS177 ILE178 ASP179 PRO180 ARG227 PHE228 SER229 PRO230 ALA277 PHE278 SER279 PHE320 ASN321 SER323
Hydroquinone 1,4‐Benzenediol	1281	−5.5	**Chain A**: TYR1787 HIS1791 VAL1795 PHE1798 GLU1799 LEU1802 LYS1805 GLN1901 ASP1902 LEU1904 ARG1905 VAL1906 LEU1907 THR1908 LEU1909 PHE1911 ASP1912 GLN1941 ALA1944 ARG1945 ARG2408 GLU2409 HIS2410 LYS2411 ASP2412 SER2413 MET2415 ALA2416 VAL2417 GLU2419 ALA2420 PHE2421 TYR2423 ASP2424 PRO2425 ARG2505
Cyclodecasiloxane	2490	−5	**Chain B**: GLN1715 GLN1718 HIS1719 VAL1721 GLN1722 THR1723 GLN1725 GLN1726 GLN1729 HIS1730 PHE1751 LEU1754 LYS1771 GLN1774 TYR1775 **Chain A**: ILE2267 ARG2270 HIS2289 ASN2293 **Chain C**: ILE260 SER262 GLY263 ASN264 GLU267 SER268 SER269 ARG270 ASP291 ASN292 LEU293 ARG295 GLU307
1‐Hexadecene	4675	−5.2	**Chain B**: ASP2276 HIS2277 LEU2278 THR2279 LEU2280 MET2281 GLN2282 LYS2283 PHE2371 PRO2372 LYS2374 GLN2540 **Chain D**: ASN46 ALA47 LEU48 GLU49 TYR62 LYS86 ASN87 ALA89 SER90 VAL91 GLU105 PRO130 ASN132 CYS133 VAL134 GLN148 THR174 SER175 ALA176 LEU224 GLN225 TRP274 GLY275 CYS276 VAL316 CYS317 LEU318
Methyl 3‐hydroxytetradecanoateTetradecanoic acid, 3‐hydroxy‐, methyl ester	3178	−5.8	**Chain A**: THR2279 MET2281 GLN2282; **Chain C**: VAL45 ASN46 ALA47 LEU48 GLU49 ALA89 SER90 VAL91 ASN132 CYS133 VAL134 CYS135 THR174 SER175 ALA176 LEU224 GLN225 CYS226 ARG227 TRP274 GLY275 CYS276 ALA288 VAL316 CYS317 LEU318 ALA319
Salicylaldehyde hydrazone Benzaldehyde, 2‐hydroxy‐, hydrazine	1281	−8	**Chain A**: VAL1795 PHE1798 GLU1799 LEU1802 LYS1805 HIS1806 GLN1901 LEU1904 ARG1905 LEU1907 THR1908 PHE1911 ASP1912 GLN1941 ARG1945 LYS2411 ASP2412 MET2415 ALA2416 GLU2419 ALA2420 PHE2421 TYR2423 ASP2424 PRO2425 ASN2494 ILE2498 ILE2501 ASN2502 ARG2505
α‐d‐Glucopyranose, 4‐*O*‐α‐d‐galactopyranosylLactose, α	3178	−8.5	**Chain A**: THR2279 MET2281 GLN2282; **Chain C**: VAL45 ASN46 ALA47 LEU48 GLU49 ILE88 ALA89 SER90 VAL91 GLY92 ASN132 CYS133 VAL134 CYS135 ILE173 THR174 SER175 ALA176 HIS177 LEU224 GLN225 CYS226 ARG227 TRP274 GLY275 CYS276 ALA277 VAL316 CYS317 LEU318 ALA319
2‐Propenoic acid, 3‐(4‐fluorophenyl)‐, ethyl ester	4675	−6.8	**Chain B**: ASP2276 HIS2277 LEU2278 THR2279 LEU2280 MET2281 GLN2282 LYS2283 GLN2540; **Chain D**: ASN46 ALA47 LEU48 GLU49 TYR62 LYS86 ASN87 ALA89 SER90 VAL91 GLU105 PRO130 ASN132 CYS133 VAL134 GLN148 THR174 SER175 ALA176 HIS177 LEU224 GLN225 CYS226 ARG227 TRP274 GLY275 CYS276 VAL316 CYS317 LEU318
î‐*N*‐Formyl‐l‐lysine	2490	−5.7	**Chain B**: PRO1688 LEU1689 PRO1690 THR1691 GLN1715 HIS1716 MET1717 HIS1719 PHE1720 GLN1722 THR1723 MET1724 GLN1726 GLN1727 **Chain A**: ARG2266 ILE2267 ARG2270 MET2271 ALA2272 GLU2285 GLU2288 HIS2289 ASN2292; **Chain C**: TYR20 ARG221 ALA240 SER268 SER269 ARG270 GLY271 TRP272 SER290 ASP291 ASN292 GLN312 LYS313 ALA314
Pentadecanoic acid, 13‐methyl‐, methyl ester	4675	−11.4	**Chain B**: THR2279 LEU2280 MET2281 GLN2282; **Chain D**: ASN46 ALA47 LEU48 GLU49 ALA89 SER90 VAL91 GLY92 PHE93 GLU105 ILE131 ASN132 CYS133 VAL134 CYS135 LEU136 GLN148 THR174 SER175 ALA176 HIS177 LEU224 GLN225 CYS226 ARG227 TRP274 GLY275 CYS276 VAL316 CYS317 LEU318
4‐Cyclohepta‐2,4,6‐trienyl‐benzoic acid	4675	−7.6	**Chain B**: THR2279 LEU2280 MET2281 GLN2282; **Chain D**: ASN46 ALA47 LEU48 GLU49 ALA89 SER90 VAL91 GLU105 ILE131 ASN132 CYS133 VAL134 CYS135 GLN148 THR174 SER175 ALA176 HIS177 LEU224 GLN225 CYS226 ARG227 TRP274 GLY275 CYS276 VAL316 CYS317 LEU318 ALA31
5,6‐Dimethoxyphthalaldehydic acid	1281	−6.9	**Chain A**: VAL1795 PHE1798 GLU1799 VAL1801 LEU1802 LYS1805 HIS1806 PHE1888 GLN1901 LEU1904 ARG1905 LEU1907 THR1908 LEU1909 PHE1911 ASP1912 TYR1913 PRO1940 GLN1941 LEU1942 ALA1944 ARG1945 ARG2197 ASP2412 SER2413 MET2415 ALA2416 VAL2417 GLU2419 ALA2420 TYR2423 ASP2424 PRO2425 ASN2494 ILE2498 ILE2501 ASN2502 ARG2505

### 5.2 Docking Analysis of Ligand Against Serine/Threonine‐Protein Kinase mTOR (PDB ID: 4JSX)

**FIGURE 5:**
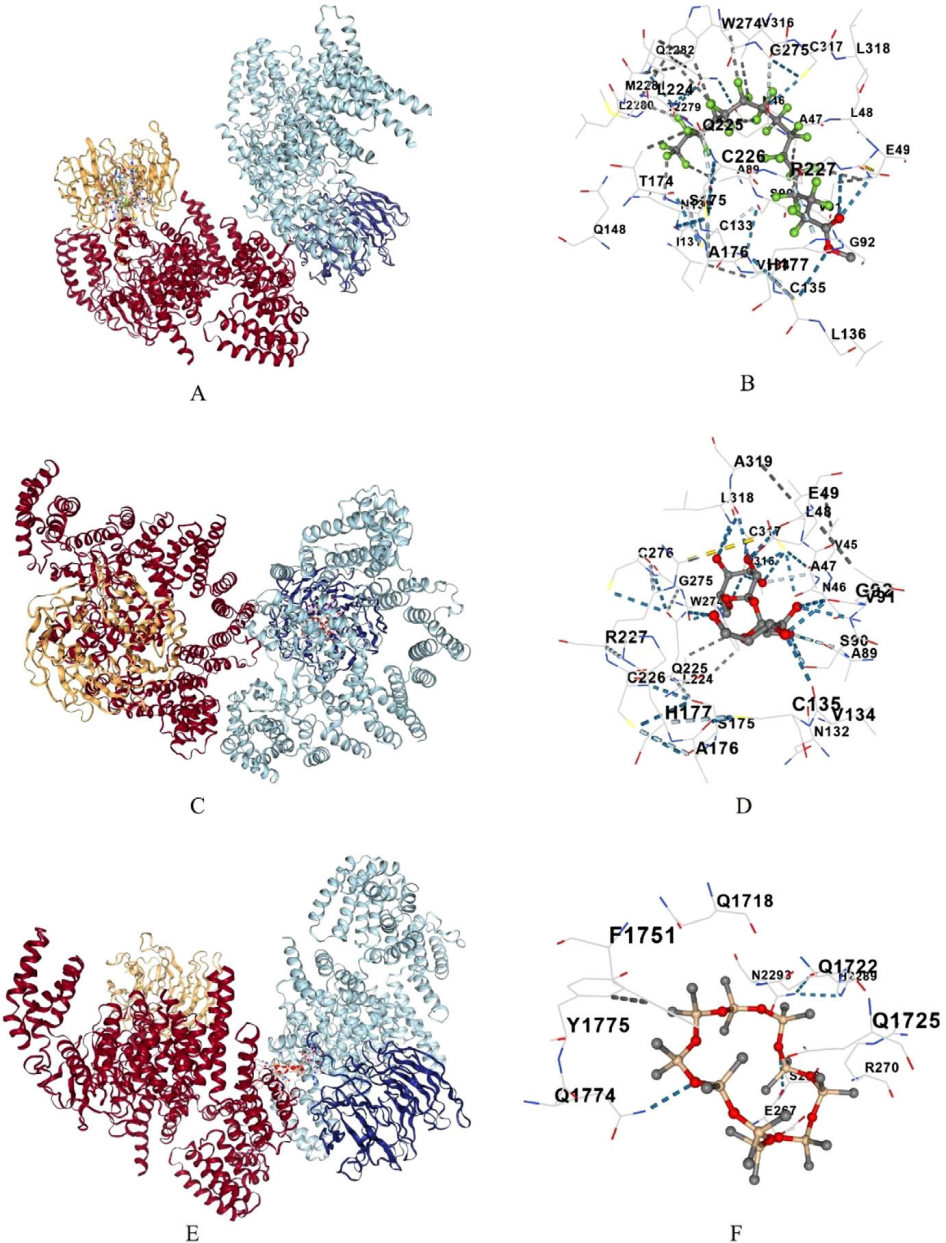
Molecular docking of identified compounds of *Si*EE against Serine/threonine‐protein kinase mTOR (PDB ID: 4JSX) (A, B) Pentadecanoic acid, 13‐methyl‐, methyl ester; (C, D) α‐d‐glucopyranose, 4‐*O*‐α‐d‐galactopyranosyl lactose; (E, F) salicylaldehyde hydrazone benzaldehyde, 2‐hydroxy‐, hydrazine.

A notable binding affinity is observed between several compounds against the target protein serine/threonine‐protein kinase mTOR (PDB: 4JSX) (Figure 5; Table 8). The identified compounds with Vina scores of −5 and above are included in the Table 8. Pentadecanoic acid, 13‐methyl‐, methyl ester exhibited the highest affinity with a Vina score of −11.4, followed by α‐d‐glucopyranose, 4‐*O*‐α‐d‐galactopyranosyllactose with a Vina score of −8.5, and salicylaldehyde hydrazone, benzaldehyde (2‐hydroxy‐, hydrazine) with a score of −8 (Figure 5; Table 8). Other compounds such as Oxazepam ditms and 2,5‐dihydroxyacetophenone, bis(trimethylsilyl) ether had Vina scores of −7.4 and −6.9, respectively (Table 8). 4‐Cyclohepta‐2,4,6‐trienyl‐benzoic acid and 2‐propenoic acid, 3‐(4‐fluorophenyl)‐, ethyl ester showed moderate binding affinity with scores of −7.6 and −6.8 (Table 8). Compounds like methyl 3‐hydroxytetradecanoate tetradecanoic acid 3‐hydroxy‐methyl ester showed the Vina scores of −5.8, while cyclopentasiloxane decamethyl and 1‐hexadecene displayed weaker binding with scores of −5.9 and −5.2 (Table 8). Other compounds like cyclodecasiloxane eicosamethyl and hydroquinone 1,4‐benzenediol had the lowest binding affinities with Vina scores of −5 and −5.5, respectively. The key amino acid residues in the protein involved in these interactions included THR2279, MET2281, GLN2282, GLU49, and CYS135, showing a range of binding sites across the protein's different chains (A, B, C, D) (Table 8).

The toxicity of *Si*EE is evaluated using the brine shrimp lethality assay, and a dose dependent toxicity is recorded. The LC_50_ value is determined to be 274.26 mg/mL, with 95% confidence limits ranging from 239.09 mg/mL (lower) to 307.44 mg/mL (upper). In addition, the LC_90​_ value is recorded as 520.59 mg/mL, indicating the concentration required to achieve 90% lethality. The chi‐square (*χ* ^2^) value of 2.300 suggested a good fit for the statistical model, confirming the reliability of the data. These findings indicated that *S. inaequalifolia* possesses moderate cytotoxic potential; this effect may result from its bioactive compounds, emphasizing the importance of further pharmacological research.

**FIGURE 6:**
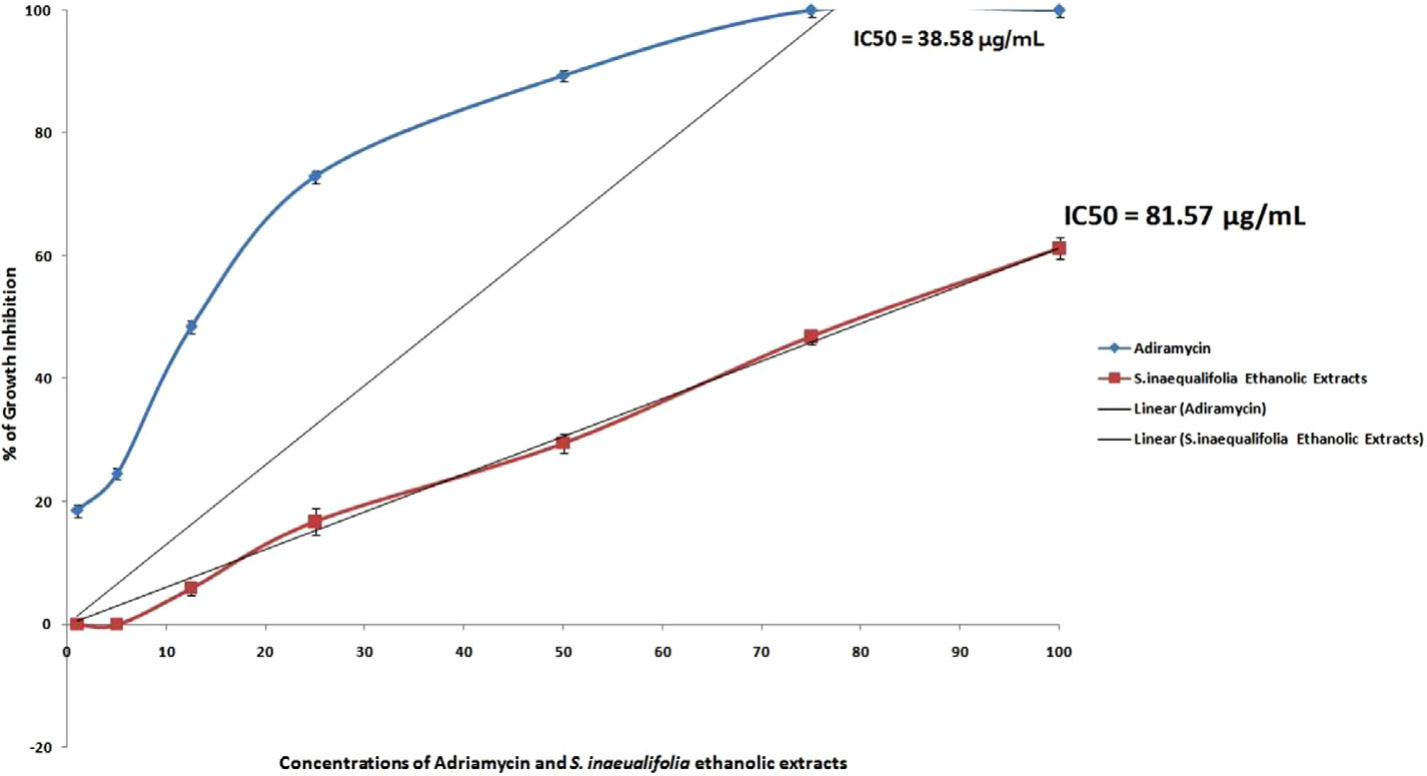
Cytotoxic activity of *Selaginella inaequalifolia* ethanolic extracts and adiramycin against MCF cell line (% of the growth inhibition ± SE).

Figure 6 showed the effect of *S. inaequalifolia* on cell line growth inhibition against MCF‐7 Cell lines at different concentrations. The IC_50_ values of *Si*EE was 81.57 µg/mL and standard adiramycin was 38.58 µg/mL (Figure 6).

## 6 Discussion

The amalgamation of GC–MS–mass spectrometry elevated the GC–MS as one of the highly efficient tool for the separation and identification of active principles from the crude extracts. In the present study, GCMS is employed to reveal the phytoprofile and chemical constituents of *Si*EE. Previous studies have employed GC–MS for the qualitative and quantitative profiling of volatile, semi‐volatile, and nonpolar compounds and bioactive principles (*Pteridium aquilinum*—[44], *Asplenium aethiopicum*—[58], *Dryopteris hirtipes*—[45], *Pteris togoensis*—[46], and *Dicranopteris linearis*—[47]). In the present study, the GC–MS analysis revealed the existence of the various bioactive principles in *Si*EE. PASS is employed to predict the biological properties of the crude extracts derived compounds and fraction of the crude extracts. Previous predictions using the PASS have identified the potential bioactivities of various medicinal plants, including *Vincetoxicum subramanii* [61], *Psidium guajava* [48], *Ficus benghalensis*, and *Ficus krishnae* [59], *Albizia lebbeck* [49], and *Andrographis paniculata* [50]. The PASS analysis suggested the medicinal properties of the *S. inaequalifolia*. The ADME and toxicity profiles of bioactive compounds from *Si*EE are assessed and revealed that several compounds have favorable drug‐likeness characteristics. 2,5‐Dihydroxyacetophenone, bis(trimethylsilyl) ether, and cyclopentasiloxane, decamethyl showed good solubility and GI absorption, while methyl 3‐hydroxy‐tetradecanoate exhibited high absorption despite CYP2D6 inhibition. However, 2,5‐dihydroxyacetophenone and methyl 3‐hydroxy‐tetradecanoate may cause drug interactions due to enzyme inhibition [51]. Oxazepam ditms and hydroquinone exhibited poor solubility or toxicity, particularly in dermal and inhalation tests. IARC [56, 57] reported that Oxazepam ditms are carcinogenic to human.

CDK2 (PDB: 4GCJ) showed the strongest binding (Vina score: −11.0 kcal/mol) with pentadecanoic acid, 13‐methyl‐, methyl ester. Other compounds like 4‐cyclohepta‐2,4,6‐trienyl‐benzoic acid and salicylaldehyde hydrazone also demonstrated significant interactions. Pentadecanoic acid, 13‐methyl‐, methyl ester as a strong mTOR (PDB: 4JSX) binder with a Vina score of −11.4 kcal/mol, comparable to known inhibitors like rapamycin and Torin1 [52]. The molecular docking studies results showed that the pentadecanoic acid, 13‐methyl‐, methyl ester as potential inhibitors of CDK2 and mTOR. Hence the outcome of the present study revealed the potential of pentadecanoic acid, 13‐methyl‐, methyl ester as CDK2 and mTOR inhibitor, warranting further investigation through molecular dynamics simulations and in vitro validation.

The median lethal concentration (LC_50_) is a crucial parameter in BSLB, representing the concentration required to kill 50% of brine shrimp larvae. Generally, an LC_50_ value below 1000 µg/mL indicates significant cytotoxic potential [64]. For instance, *Woodfordia fruticosa* exhibited moderate cytotoxicity with LC_50_ of 763.34 µg/mL [54], while *Plectranthus barbatus* root extracts demonstrated high cytotoxicity with an LC_50_ of 40.07 µg/mL (Lawi et al. 2018) [55]. These findings suggest that different plant species, and even different parts of the same plant, can exhibit significantly diverse cytotoxic profiles. The observed LC_50_ value below 1000 µg/mL indicates that *Si*EE possess significant cytotoxic potential [64]. The chi‐square (χ^2^) value of 2.300 suggests a reliable dose‐response relationship. Compared to the aforementioned medicinal plants, *Si*EE exhibit extremely low cytotoxicity, making them a safer alternative for pharmacological applications that require minimal toxicity.

Cytotoxic properties of the studied *S. inaequalifolia* might be due to the toxic compounds present in the extracts. *S. inaequalifolia* is found to be most effective with LC_50_ value 274.26 mg/mL. Cytotoxic potentials of *Si*EE are examined against MCF‐7 breast cancer cell lines. The cell growth inhibition was directly proportional to the concentration of the *Si*EE. The IC_50_ values of *Si*EE was 81.57 µg/mL and standard adiaramycin was 38.58 µg/mL (Figure 6). Typically cytotoxic activity showed IC_50_ values in the range of < 1 to 100 µg/mL The crude *Si*EE showed a good growth inhibition with IC_50_ values 81.57 µg/mL. It leads to identification of novel antitumor and anticancer agents in the *Si*EE.

## 7 Conclusion

This study highlights the efficiency of GC–MS in identifying bioactive compounds from *Si*EE, revealed several phytochemicals with potential pharmacological applications. The ADME and toxicity profile analysis identified pentadecanoic acid, 13‐methyl‐, methyl ester as a strong CDK2 and mTOR inhibitor, suggesting anticancer potential. The brine shrimp lethality assay (LC_50_: 274.26 mg/mL) indicated low cytotoxicity, while MCF‐7 breast cancer cell line studies (IC_50_: 81.57 µg/mL) showed promising anticancer activity. These findings support *S. inaequalifolia* as a potential source of therapeutic agents, warranting further molecular and clinical investigations. Further studies on the isolated active principles may bring out a plant based anticancer agents.

## Conflicts of Interest

The authors declare no conflicts of interest.

## Data Availability

The authors have provided all the experimental data.
